# Supplementary material for: Identification and functional analysis of non-coding regulatory small RNA FenSr3 in Bacillus amyloliquefaciens LPB-18
Source: PeerJ. 2023 May 15;11:e15236. doi: 10.7717/peerj.15236 (PMC10194069; doi:10.7717/peerj.15236)
Supplement: Supplemental Information 4 [file peerj-11-15236-s004.zip › KO/CK-vs-T1_map/map01503.html]

KEGG PATHWAY: Cationic antimicrobial peptide (CAMP) resistance - Reference pathway


|  |  |
| --- | --- |
| **Cationic antimicrobial peptide (CAMP) resistance - Reference pathway** |  |

[
Pathway menu
| Organism menu
| Pathway entry
| Show description
| User data mapping
]

Cationic antimicrobial peptides (CAMPs) play an important role in host defense against microbial infection and are key components of the innate immune response. These are found among all classes of life ranging from prokaryotes to humans. In addition to the natural peptides, thousands of synthetic variants have been produced. CAMPs weaken the integrity of the bacterial inner and outer membranes and subsequently kill bacterial cells. On the other hand, bacteria have developed a number of mechanisms against CAMPs. These resistance mechanisms include decreased affinity to CAMPs by substitution of anionic cell surface constituents with cationic molecules; biosynthesis and crosslinking of cell envelope components; external trapping mechanisms that bind or neutralize the CAMPs by direct secretion of proteins, or the release of CAMPs binding molecules from the host cell surface; membrane efflux pumps; and production of peptidases.

|  |  |  |
| --- | --- | --- |
| Reference pathway | 184% 150% 122% 100% 82% 67% 55% | 图片下载 |
